# Neuromorphic Engineering Needs Closed-Loop Benchmarks

**DOI:** 10.3389/fnins.2022.813555

**Published:** 2022-02-14

**Authors:** Moritz B. Milde, Saeed Afshar, Ying Xu, Alexandre Marcireau, Damien Joubert, Bharath Ramesh, Yeshwanth Bethi, Nicholas O. Ralph, Sami El Arja, Nik Dennler, André van Schaik, Gregory Cohen

**Affiliations:** International Centre for Neuromorphic Systems, The MARCS Institute for Brain, Behaviour, and Development, Western Sydney University, Penrith, NSW, Australia

**Keywords:** neuromorphic engineering, benchmarks, event-based systems, DAVIS, DVS, ATIS, audio, olfaction

## Abstract

Neuromorphic engineering aims to build (autonomous) systems by mimicking biological systems. It is motivated by the observation that biological organisms—from algae to primates—excel in sensing their environment, reacting promptly to their perils and opportunities. Furthermore, they do so more resiliently than our most advanced machines, at a fraction of the power consumption. It follows that the performance of neuromorphic systems should be evaluated in terms of real-time operation, power consumption, and resiliency to real-world perturbations and noise using task-relevant evaluation metrics. Yet, following in the footsteps of conventional machine learning, most neuromorphic benchmarks rely on recorded datasets that foster sensing accuracy as the primary measure for performance. Sensing accuracy is but an arbitrary proxy for the actual system's goal—taking a good decision in a timely manner. Moreover, static datasets hinder our ability to study and compare closed-loop sensing and control strategies that are central to survival for biological organisms. This article makes the case for a renewed focus on closed-loop benchmarks involving real-world tasks. Such benchmarks will be crucial in developing and progressing neuromorphic Intelligence. The shift towards dynamic real-world benchmarking tasks should usher in richer, more resilient, and robust artificially intelligent systems in the future.

## 1. Introduction

Despite the significant strides made in neuromorphic engineering in recent years, the field has not yet seen widespread industrial or commercial adoption. There is clearly difficulty in translating the research output of the field into real-world and commercially successful applications. Neuromorphic engineering has individually demonstrated many significant and valuable concepts, evidenced by dedicated large-scale neuromorphic processors (Davies et al., 2018), power-efficient analogue neuron circuits (Chicca et al., 2014; Moradi et al., 2018), on-chip and local unsupervised learning circuitry (Qiao et al., 2015), scalable parallel message-passing architectures (Furber, 2016), and retina-inspired and compressed visual sensing (Lichtsteiner et al., 2008). There are also active research and commercialisation efforts in applications of this research, including in Event-based Space Situational Awareness (Cohen et al., 2019), autonomous vehicle sensors (Perot et al., 2020; Gehrig et al., 2021), and for home security monitoring (Park et al., 2019; Samsung, 2020). However, the field struggles to integrate, build upon, and convey these successes to the wider engineering and scientific community.

This article examines potential reasons for this slow dissemination by assessing the role that datasets, benchmarking problems, and comparative metrics play in presenting neuromorphic engineering to existing scientific communities. Adhering to existing benchmarking metrics, designed for fundamentally different processing and sensing systems, may limit our ability to report and, perhaps more importantly, to describe the performance and advantages of neuromorphic systems. Additionally, the ubiquity of such metrics complicates the development of new approaches to tackling existing problems with novel solutions. This is especially prevalent when moving away from uniformly sampled sensing and synchronised processors.

Progress in conventional computer vision and machine learning has been built upon datasets and static problems. The most significant strides in computer vision and deep neural networks were spurred by the *ImageNet moment* (Krizhevsky et al., 2017) and the rise of data-driven systems (Torralba and Efros, 2011), leading to some truly astonishing capabilities, from the ability to achieve human-like (and even super-human) levels of performance under ideal viewing conditions on certain vision tasks (He et al., 2016; Geirhos et al., 2017), to the unsettling ability to realistically replace faces and people in high-definition video (Wang et al., 2021). However, such cutting-edge data-driven systems require unprecedentedly large datasets that have only become feasible in terms of size and required computation starting with the release of ImageNet in 2012 and the advent of high-performance computing centres. These data-driven approaches are unlikely to scale with increasing task complexity. The corresponding networks ingesting this data have grown vast in size and scale. Large datasets become difficult to distribute and test against and even more difficult to collect. Only a handful of organisations possess the resources required to collect and generate the cutting-edge datasets used at the forefront of deep learning. Furthermore, immunity to variable and degraded viewing conditions are still a problem that static datasets do not tackle efficiently and closed-loop benchmarks are better suited to test these conditions.

Larger datasets have enabled researchers to train ever-larger networks, but, importantly, have also provided a meaningful way to compare different algorithms and approaches. This has driven researchers to optimise and push the limits of the technologies and algorithms through a mutually understood and quantifiable way of measuring success. Novel datasets and benchmarks will not only push model and algorithmic complexity, but also implicitly advance our understanding of distributed, parallel, and even neural computation.

Neuromorphic engineering has naturally followed a similar trajectory, both through the conversion of existing datasets to a neuromorphic format (Orchard et al., 2015a) and through the collection and creation of new datasets (Perot et al., 2020; Gehrig et al., 2021). The growth of neuromorphic computing has further driven the need for suitable neuromorphic benchmarks to showcase the utility of its approaches to artificial intelligence. Similar to conventional machine learning, this demand has led to the rise and proliferation of static neuromorphic datasets and, similarly, these have been instrumental in the field's advancement and growth.

However, our paper will detail how these approaches may actually be constricting the ability of neuromorphic engineering to tackle real-world problems in novel ways using approaches that embody and showcase the unique benefits of a fundamentally different way of operation. We discuss the history of neuromorphic benchmarking (see Section 1.1) and highlight the advantages and implications of sensing and processing in the context of closed-loop control systems (see Section 2). We further provide an overview of existing open-loop datasets, discuss in greater detail their downsides (see Section 3), and then apply the same analysis towards existing open-loop neuromorphic benchmarks (see Section 3.1).

After a brief overview and discussion of existing closed-loop conventional benchmarks (see Section 3.2) and simulation environments available to create new closed-loop benchmarks (see Section 3.3), we describe our efforts in designing and developing a new generation of physically embedded, closed-loop neuromorphic benchmarks (see Section 4). We finish with concluding remarks for future developments of closed-loop benchmarks to bootstrap the next generation of artificial and neuromorphic intelligence.

### 1.1. History of the Analysis of Neuromorphic Benchmarks

The neuromorphic community has long recognised the importance of datasets and their potential limitations, which led to a special research topic in Frontiers in Neuroscience: Neuromorphic Engineering in 2015 devoted specifically to neuromorphic benchmarking and challenges^1^. The proposal for the topic describes a situation not dissimilar to the current state of neuromorphic engineering in terms of the ability to make meaningful and representative comparisons of neuromorphic systems, both to one another and conventional systems. The papers published in that research topic provided a thorough overview of the existing efforts to create benchmarking approaches and included papers focusing on domain-specific and modality-specific sets of requirements and needs.

In fact, Stewart et al. (2015) directly addressed the need for closed-loop benchmark tasks in neuromorphic engineering, describing a closed-loop benchmark as a two-way interaction task in which the “output of the neuromorphic hardware influences its own future input” (Stewart et al., 2015). Highlighting the challenges involved in providing closed-loop tasks in place of static datasets, the authors suggested that this can only be accomplished by either providing a fully-specified physical system or providing a software simulation of the system. Building upon these ideas, this article summarised the existing simulators and related problems, highlighting the shortcomings of simulators and the difficulty in translating these into real-world applications and strongly motivating the need for real-world physical systems.

In addition, Tan et al. (2015) provided a thorough summary of the efforts to benchmark neuromorphic vision systems and outlines some of the lessons learned in creating and using the available datasets. Core to the arguments, in this article, the authors introduce the problems encountered when using static images with neuromorphic vision sensors, highlighting that the type of static object recognition problems found in conventional computer vision has no direct parallel in biology and therefore are not a task that biological systems have evolved to tackle (Tan et al., 2015). Contributing to this point, this article also stresses that neuromorphic vision datasets should be as representative of the real-world as possible. As our paper seeks to motivate, the move to real-world benchmarking tasks will inherently solve this problem.

The discussion around the development of the Poker-DVS and MNIST-DVS datasets by Serrano-Gotarredona and Linares-Barranco (2015) also provides valuable insight into the historical reasons contributing to the reliance on datasets in the neuromorphic community. They point to the difficulty in obtaining neuromorphic hardware as a driving factor in the production of datasets that allow researchers to explore neuromorphic techniques without having physical access to scarce hardware resources. Whilst the supply and dissemination of neuromorphic sensors has drastically improved, the point remains a valid one, and there is still a strong need for neuromorphic datasets to enable access to the technologies.

Beyond the historical perspective, the authors also point out that the original poker dataset required manual curating due to noise, card edges, and the numbers on the cards. Their automated tracking method struggled with these factors, requiring annotations by hand to produce correctly labelled data. This highlights both the difficulty in acquiring large volumes of labelled data, and the question of inadvertently injecting additional context into the problem through factors such as labelling bias.

### 1.2. Promises of Neuromorphic Systems

To overcome the limitations of existing neuromorphic benchmarks, we argue that performance of neuromorphic systems should directly be evaluated based on latency, power consumption, and task-specific control metrics, rather than on a plain and static accuracy metric. This move inherently requires closed-loop sensing and processing, which in turn favours highly recurrent and feedback-heavy algorithms and architectures. Predictive algorithms naturally result, since for an agent to make an informed decision and react appropriately in a given environment, the past and present estimates of the state of said environment hardly matter. What does matter is the agent's expectation of future states, i.e., how the environment is going to change (Davies, 2019).

Closed-loop benchmarks require algorithms to holistically optimise for real-world constraints and power consumption while operating in real-time. Closed-loop benchmarks also require the ability to respond appropriately to ambiguous and partial inputs from an uncontrollable noisy and dynamic environment. Hence, we anticipate that designing algorithms for such tasks will lead to richer, more advanced, resilient, and truly intelligent artificial systems inspired by their biological counterparts. The physically embedded nature of biological processing, and the associated physical size, weight, power, and speed limitations that come with it, are fundamental aspects of the operation of such systems and cannot be treated as afterthoughts to be simulated or optimised in the final development phase.

Inspired by biological sensory-processing systems, the neuromorphic sensing and processing paradigm targets these requirements by providing resilient, parallel, asynchronous, and highly distributed sensory-processing solutions (Mead, 1990; Liu and Delbruck, 2010; Hamilton et al., 2014). The resulting neuromorphic processors are non-Von Neumann computing architectures that feature local learning mechanisms and are capable, when combined with neuromorphic sensors, of time-continuous, asynchronous and distributed information processing, with high power efficiency than their conventional clock-based counterparts (Thakur et al., 2019).

The gravitation of the neuromorphic community towards machine learning-like datasets is understandable, since the generation of alternative closed-loop datasets is at once challenging and very resource-intensive while simultaneously lacking the legitimacy of established large scale open-loop machine learning-like benchmarks (Grother, 1995; Jia Deng et al., 2009; Geiger et al., 2013; Xu et al., 2020). Novel embedded closed-loop benchmarks, however, will spur the development of closed-loop sensing, dynamic processing, and decision making systems, which is where neuromorphic computing has the greatest potential for providing advances in technology and computational models.

## 2. Different Styles of Sensing

**Figure 1:**
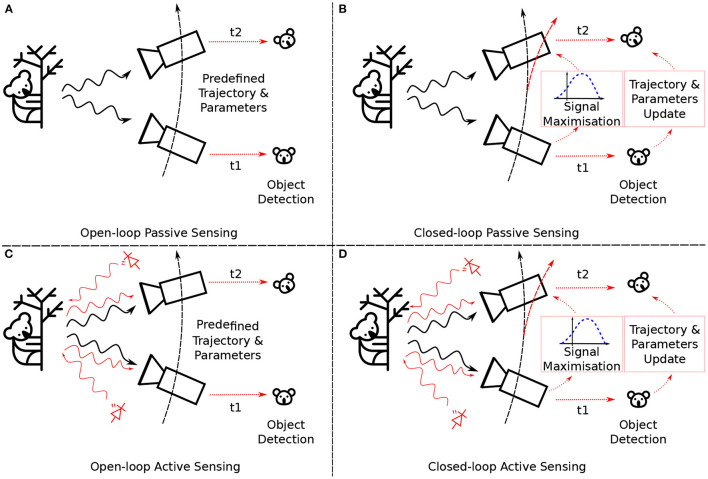
Different modes of sensing. Sensing and consequently processing of sensory information can be divided into passive **(top, A and B)** vs. active **(bottom, C and D)**, as well as open- **(left, A and C)** vs. closed-loop **(right, B and D)** sensing. Open-loop passive sensing **(A)** is the most prevalent form of acquiring information about the environment and subsequently using this information, e.g., to classify objects. Advantages of this approach include the one-to-one mapping of inputs and outputs and the readily available optimisation schemes that obtain such a mapping. Examples for open-loop passive sensing include surveillance applications, face recognition, object localisation, and most conventional computer vision applications. While the environment and/or the sensor could move, the trajectory itself is independent of the acquired information. Open-loop active sensing **(C)** is characterised by injecting energy into the environment. The acquired data is a combination of information emitted by the environment itself (black arrow) and the resulting interaction of the signal emitted by the sensor with the environment (red arrow). Prime examples of this sensing approach are LiDAR (LiDAR), RADAR, or SONAR. In the open-loop setting, the acquired information is not used to change parameters of the sensor itself. The closed-loop passive sensing strategy **(B)** is most commonly found in animals, including humans. While energy is solely emitted by the environment, the acquired information is used to actively change the relative position of the sensor (e.g., saccadic eye movements) or alter the sensory parameters (e.g., focus). This closed-loop approach utilises past information to make informed decisions in the future. The last sensing category is active closed-loop sensing **(D)** where the acquired information is used to alter the positioning and configuration of the sensor. Bats (Griffin, 1958; Fenton, 1984) and weakly electric fish (Flock and Wersäll, 1962; Hofmann et al., 2013) are prime examples from the animal kingdom that exploit this sensing style, but also artificial systems, such as adaptive LiDAR, use acquired information about the environment to perform more focused and dense information collection from subsequent measurements.

Sensors, irrespective of their sensing modality, can be classified into two distinct categories: passive sensors and active sensors. Passive sensor strategies do not emit energy into the environment when acquiring samples or data (see Figure 1, top row). A common example is found in autonomous systems, in which conventional image sensors employ a passive sensing approach to detect and process stimuli scattered by the immediate environment (Rees, 1990). In contrast, active sensors emit energy directly into their environment to elicit data, sampling a composition of the interactions of the actively emitted energy on the environment and any scattered energy already present in the environment (see Figure 1, bottom row). Autonomous systems may also employ active sensing regimes, such as RADAR and LiDAR, to parse their environment by instead varying sensor characteristics based upon the global state or by acting upon the immediate environment (Gini and Rangaswamy, 2008).

Sensor strategies can also be split by whether the control of the sensor is influenced by the output of the sensor. Moving the sensor in response to its output is also sometimes called active sensing^2^, but here we adopt the term closed-loop sensing for this mode of operation to avoid confusion, and open-loop sensing for the mode where the sensor output has no impact on the sensor itself. In open-loop systems, the sensor is simply a source of data for the rest of the system, allowing for very simple sensor designs (see Figure 1, left column). Closed-loop systems integrate the sensor far more deeply into the system, and aspects of the sensor are actively modified as a function of its output to increase the relevant information in the sensor's output (see Figure 1, right column). Closed-loop systems are more complicated to design but offer the potential to extract far more task-relevant information from the sensor.

**Figure 2:**
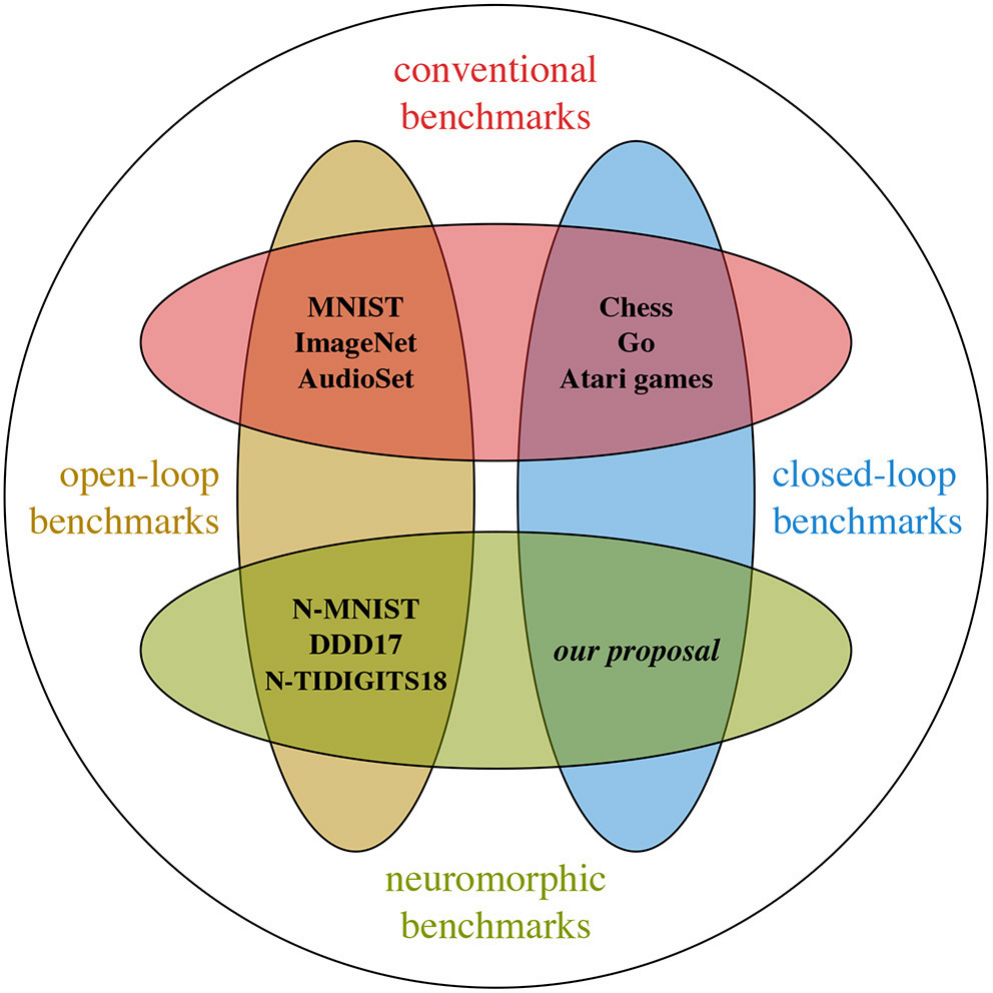
Existing datasets and benchmarks fall into two categories: open-loop benchmarks, or datasets, and closed-loop benchmarks. Supervised machine learning relies mostly on the first category, whereas reinforcement learning requires the second. Most existing neuromorphic engineering benchmarks fall in the first category. This article pleads in favour of closed-loop neuromorphic benchmarks.

These two ways to categorise sensors are not mutually exclusive, and indeed there exist closed- and open-loop strategies for both active and passive systems (see Figure 2). The passive and active sensing strategies can both benefit greatly from a closed-loop methodology, especially when an internal model of the system is used to produce informed decisions to update sensor settings and model parameters. Practical examples of such systems include the closed-loop passive sensing techniques of stimulating contrast detection in event-based vision sensors with ego-motion, trading temporal resolution for spatial resolution (Yousefzadeh et al., 2018; D'Angelo et al., 2020).

Open-loop systems have the advantage of simplicity, in terms of their design and in terms of the data that they produce. By definition, open-loop sensing does not feature a feedback mechanism and therefore acquired samples have no effect on the next sample acquired by the sensor. As the sensor can be treated solely as a static source of information, recorded datasets can easily be shared with the research community, allowing different algorithms to be compared without the need to replicate the interactions between the system and the sensor. This greatly simplifies the creation and the use of open-loop datasets, as no sensor state information needs to be known or stored. This simplicity, however, imposes limits on the nature of the problems being tackled. Problems are often carefully chosen, or restricted, to enable the use of an open-loop sensor (for a non-exhaustive list of existing open-loop datasets see Section 3). Such open-loop sensing approaches and their resulting datasets, however, limit the real-world applicability of an algorithm as information that could be beneficial to adjust to the environment is irreversible lost.

Systems for real-world problem solving such as autonomous driving (Bojarski et al., 2016) and process control (Firoozian, 2014) generally require algorithms with feedback mechanisms to proactively sample the environment and act accordingly. Potential feedback actions include changing the sensor position, the sensor configuration, or some aspect of the interface between the sensor and the environment. Adding a mechanism of feedback allows a system to observe the result of its interaction with the environment when solving compound real-world problems (Åström and Murray, 2010). With the inclusion of some element of (dynamic) memory capacity, these systems can be extended to achieve a degree of statefulness, using the recurrent nature of the system feedback to build an internal model of the surrounding environment (Rao and Ballard, 1999; Friston, 2010; Rasmussen et al., 2017; Hogendoorn and Burkitt, 2018; Keller and Mrsic-Flogel, 2018).

The stateful memory capacity inherent in closed-loop systems is partially determined by the dimensionality of the feedforward signal, but primarily determined by the dimensionality of both feedback and recurrent pathways. Here, neuromorphic sensory-processing systems are of special interest due to their continuous and implicit representation of time in sensing and processing, thus increasing the resolution of their temporal dimension for all three information pathways (feedforward, feedback, and recurrent). This has the consequence, especially in closed-loop systems, that signals can be asynchronously distributed without the need for a centralised clock.

The path going forward towards machine intelligence, especially for neuromorphic technology, is not merely a substitution of neuromorphic sensors for conventional sensors, but instead, the creation of complete embedded systems that emulate the performance and constraints of their biologically inspired origins. To address this gap and to progress with closed-loop benchmarking, we propose to build benchmarks that are physically embedded and require models operating in biological real-time. This approach provides the benchmark with an objective that inherently includes some form of decision making and action selection. These benchmarks would additionally feature sensory-motor systems that are subject to real-world fluctuations and noise, which the models would need to deal with.

## 3. Existing Benchmarks

The development of engineering systems, whether neuromorphic or otherwise, is driven by empirical studies on specific tasks or problems. The quality of a solution is measured with a benchmark—that is, a well-defined task associated with a test. The test yields a numerical score which can be used to compare solutions.

Complex problems in science and engineering are usually split into smaller ones; the so-called divide-and-conquer approach (Dustdar et al., 2020). Accordingly, benchmarks are generally designed for specific sub-problems rather than real-world tasks, with the underlying assumption that solving sub-problems is integral to tackling real-world tasks. Datasets are a simple yet effective way to implement this strategy. Labelled real-world data makes for a reasonably neutral ground truth, which can be used to estimate an algorithm's accuracy, i.e., the distance to the ground truth, with respect to an agreed upon metric. This approach yields well-defined evaluation standards that facilitate comparison between methods, and encourage competition between researchers. For example, the NIST database (Grother, 1995) provides an objective measure of individual character recognition as a means to tackle handwriting recognition. It also serves as a good entry point for more complex machine learning problems (LeCun et al., 1998). Being valid representatives of a broader class of useful problems is a sought after feature for sub-problems (Davies, 2019).

Unfortunately, the divide-and-conquer approach has several shortcomings hindering our ability to design neuromorphic systems that tackle real-world tasks. First, tackling sub-problems marginalises concerns that are only meaningful when considering real-world systems, notably power consumption and latency. It also encourages accuracy maximisation in arbitrary parts of the system, even if that accuracy may not be needed to solve the associated real-world task.

As far as neuromorphic engineering is concerned, the vast majority of existing benchmarks are open-loop (see Section 3.1 for critical review). Thus, there is no standard way to evaluate a closed-loop neuromorphic system's performance, latency or power consumption, even though neuromorphic engineering is well-suited to the design of such systems (Stewart et al., 2015).

**Figure 3:**
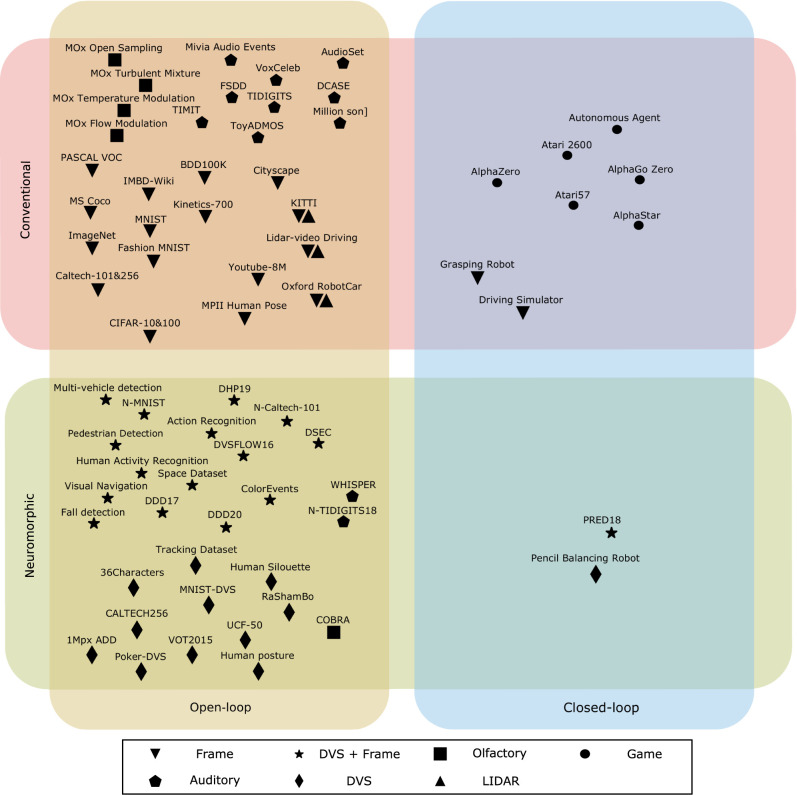
Overview of existing open- and closed-loop datasets and benchmarks for conventional time-varying and neuromorphic time-continuous approaches to machine intelligence. Distribution of high-end challenges according to the research field (neuromorphic/conventional), their interactions with the environment (open- and closed-loop), and the sensing modality. Downward triangle: conventional frame-based cameras; Diamond: neuromorphic event-based cameras; Star: Combination of conventional frame-and neuromorphic event-based cameras; Pentagon: auditory sensors; Square: olfactory sensors; Triangle: LiDAR sensors; Circles: abstract games operating directly on machine code. Further details are provided in Tables 1, 2. While not being completely exhaustive, this figure underlines the gravitation of both machine and neuromorphic intelligence community towards open-loop datasets. In order to showcase and truly contribute to the advancement of machine intelligence, the neuromorphic community needs to focus their efforts on creating closed-loop neuromorphic benchmarks that are physically embedded in their environment and thus dictate a hard power and execution time constraint. While the physical set-ups in Moeys et al. (2016) and Conradt et al. (2009) could have formed the basis of closed-loop benchmarks, they were not developed as such. In Moeys et al. (2016), the set-up was used to generate an open loop static dataset and in Conradt et al. (2009), no dataset was generated. In contrast, the benchmarks advocated here would be available as physical experimental set-ups that can be accessed by the community for algorithm testing.

Datasets are not the only type of benchmark. The Reinforcement Learning (RL) community relies on (simple) tasks that encompass both perception and action, such as Chess (Silver et al., 2018), Atari games (Mnih et al., 2013), or Go (Silver et al., 2017). The task itself is used as benchmark, therefore, the score is directly related to the intended outcome of the system, rather than being an arbitrary proxy (see Section 3.2 for short review). Much like conventional open-loop benchmarks, existing closed-loop benchmarks cannot be used directly by neuromorphic engineering. The sensing modalities are fundamentally incompatible, and noise-free data is not representative of the output of neuromorphic sensors. Nevertheless, using simple yet complete problems as benchmarks is an idea that can be translated to neuromorphic engineering. Figure 3 illustrates our view of the current situation and shows that closed-loop neuromorphic benchmarks are heavily underrepresented.

### 3.1. Neuromorphic Open-Loop Datasets

The fundamental difference between conventional sensors and neuromorphic event-based sensors is in the way the signal of interest is sampled. While the former sampling approach uses discrete and fixed time intervals to synchronously sample the signal of interest, i.e., Riemann sampling (Åström and Bernhardsson, 2002), the latter approach uses only the relative change in signal amplitude to trigger the asynchronously reporting of events, i.e., Lebesque sampling (Åström and Bernhardsson, 2002).

To still be able to utilise the tremendous effort invested by the machine learning and machine intelligence community to construct open-loop datasets, they would need to be converted to comply with neuromorphic sensory-processing systems. In order to convert existing frame-based open-loop datasets into a spike- or event-based one, the pixel intensities, in case of a vision dataset, are used to calculate a Poisson distributed spike train (Orchard et al., 2015b; Cohen et al., 2018), or to calculate the time-to-first spike (Masquelier, 2012).

Alternatively, event-based sensors have been used directly to recreate existing open-loop datasets for handwritten digit recognition (Diehl and Cook, 2015; Orchard et al., 2015a; Cohen et al., 2018), object classification (Orchard et al., 2015a; Serrano-Gotarredona and Linares-Barranco, 2015; Cohen et al., 2018), autonomous driving (Binas et al., 2017; Hu et al., 2020), pedestrian detection (Miao et al., 2019), pose estimation (Mueggler et al., 2017; Calabrese et al., 2019), spoken digit classification (Anumula et al., 2018), or speaker identification (Ceolini et al., 2020) (please refer to Figure 3 or Tables 1, 2 for a more complete listing of existing datasets and benchmarks).

**Table 1:** Conventional Benchmark Datasets for various sensor modalities.

	**Name**	**Sensor**	**Source**
	IMDB-Wiki	Frames (-)	Rothe et al. (2018)
	Kinetics-700	Frames (-)	Kay et al. (2017); Smaira et al. (2020)
	MS Coco	Frames (-)	Lin et al. (2014)
	Pascal VOC	Frames (-)	Everingham et al. (2015)
	MPII Human Pose	Frames (-)	Andriluka et al. (2014)
	YouTube-8M	Frames (-)	Abu-El-Haija et al. (2016)
	MNIST	Frames (28x28)	LeCun et al. (1998)
	Fashion-MNIST	Frames (28x28)	Xiao et al. (2017)
	CIFAR-10 & -100	Frames (32x32)	Torralba et al. (2008)
	Caltech-101 & -256	Frames (32x32)	Fei-Fei et al. (2004, 2006); Griffin et al. (2007)
Open-Loop	IMageNet	Frames (482x418)	Jia Deng et al. (2009)
	Cityscapes	Frames (1600x1200) & HDR	Cordts et al. (2016)
	KITTI	Frames (1382x512) & LiDAR	Geiger et al. (2013)
	BDD100K	Frames (720x1280)	Yu et al. (2020)
	Oxford RobotCar	Frames (1280x960) & LiDAR	Maddern et al. (2017)
	LiDAR-Video Driving	Frames (1920x1080) & LiDAR	Chen et al. (2018b)
	FSDD	Microphone (1 Ch. @ 8kHz)	Jackson et al. (2018)
	AudioSet	Microphone (-)	Gemmeke et al. (2017)
	TIDIGITS	Microphone (1 Ch. @ 20 kHz)	Leonard and Doddington (1993)
	TIMIT	Microphone (1 Ch. @ 16 kHz)	Garofolo et al. (1993)
	VoxCeleb	Microphone (1 Ch. @ 16 kHz)	Nagrani et al. (2020)
	DCASE 2020	Microphone (Mult. Ch. @ 24 kHz)	Politis et al. (2020); Heittola et al. (2020)
	ToyADMOS	Microphone (4 Ch. @ 48 kHz)	Koizumi et al. (2019)
	Mivia Audio Events	Microphone (1 Ch. @ 32 kHz)	Foggia et al. (2015)
	Million Song	Microphone (1-2 Ch. @ 22-44 kHz)	Bertin-Mahieux et al. (2011)
	MOx Open Sampling	Olfaction (9x8 Ch. @ 100 Hz)	Vergara et al. (2013)
	MOx Turbulent Mixture	Olfaction (8 Ch. @ 50 Hz)	Fonollosa et al. (2014)
	MOx Temperature Modulation	Olfaction (14 Ch. @ 3.5 Hz)	Burgués et al. (2018)
	MOx Flow Modulation	Olfaction (16 Ch. @ 25 Hz)	Ziyatdinov et al. (2015)
Closed-Loop	Atari57	Game (-)	Badia et al. (2020)
	Atari 2600	Game (210x160)	Bellemare et al. (2015)
	AlphaGo Zero	Game (-)	Silver et al. (2017)
	AlphaZero	Game (-)	Silver et al. (2018)
	AlphaStar	Game (-)	Vinyals et al. (2019)
	Autonomous Agent	Simulation (-)	Jordan et al. (2019)
	Driving simulator	Simulation (160x320)	Santana and Hotz (2016)
	Grasping Robot	Frames (-)	Stewart et al. (2015)

**Table 2:** Neuromorphic Benchmark Datasets for various sensor modalities.

	**Name**	**Sensor**	**Source**
	Pedestrian detection	DAVIS 346 (346x240)	Miao et al. (2019)
	Space Dataset	DAVIS 240 (240x180) & ATIS (304x240)	Cohen et al. (2019)
	DVSFLOW16	DAVIS 240 (240x180)	Rueckauer and Delbruck (2016)
	Visual navigation	DAVIS 240 C (240x180)	Barranco et al. (2016)
	Action recognition	DAVIS 346 (346x240)	Miao et al. (2019)
	Multi-vehicle detection	DAVIS 346 (346x240)	Chen et al. (2018a)
	DHP19	DAVIS 346 (346x240)	Calabrese et al. (2019)
	Fall detection	DAVIS 346 (346x240)	Miao et al. (2019)
	DDD17	DAVIS 346 B (346x240)	Binas et al. (2017)
	DDD20	DAVIS 346 B (346x240)	Hu et al. (2020)
	ColorEvents	ColorDAVIS 346 (346x240)	Scheerlinck et al. (2019)
Closed-Loop	1Mpx Automotive Detection Dataset	High-resolution EBC (Finateu et al., 2020) (1280–720)	Perot et al. (2020)
	DSEC	PPS3MVCD (640x480)	Gehrig et al. (2021)
	RaShamBo	DVS (64x64)	Lungu et al. (2017)
	36Characters	DVS (128x128)	Orchard et al. (2015b)
	MNIST-DVS	DVS (128x128)	Serrano-Gotarredona and Linares-Barranco (2015)
	Poker-DVS	DVS (128x128)	Serrano-Gotarredona and Linares-Barranco (2015)
	VOT2015	DVS (128x128)	Hu et al. (2016)
	Tracking Dataset	DVS (128x128)	Hu et al. (2016)
	UCF-50	DVS (128x128)	Hu et al. (2016)
	CALTECH256	DVS (128x128)	Hu et al. (2016)
	Human silhouette	DVS (128x128)	Pérez-Carrasco et al. (2013)
	Human posture	DVS (128x128)	Zhao et al. (2015)
	N-Caltech101	ATIS (304x240)	Orchard et al. (2015a)
	N-MNIST	ATIS (304x240)	Orchard et al. (2015a)
	Human activity recognition	ATIS (346x240)	Pradhan et al. (2019)
	N-TIDIGITS18	DAS (64x2x4)	Anumula et al. (2018)
	WHISPER	Microphone (16)	Ceolini et al. (2020)
	COBRA	Olfaction (-)	Schneider and Schneider (2003); Schmuker and Schneider (2007)
Closed-Loop	PRED18	DAVIS240C (240x180)	Moeys et al. (2016)
	Pencil Balancing Robot	DVS (128x128)	Conradt et al. (2009)
			
			
			
			

### 3.2. Conventional Closed-Loop Benchmarks

In closed-loop systems, contrary to open-loop ones, a sensor or agent is continuously receiving sensory stimuli from the environment (either time-varying or time-continuous). This sensory information is processed and ultimately used to either select an action or provide motor command signals that manipulate the environment or move the agent/sensor within it. Closed-loop interaction with the environment, as used in RL, alleviates the need to collect and hand-annotate large amount of data, as the agent learns online and based on partial information (Shalev-Shwartz, 2011) to maximise a reward.

The OpenAI gym environments (Brockman et al., 2016) provide a rich collection of curated closed-loop environments such as Atari games^3^, and continuous control tasks for robotic applications^4^. The OpenSim-RL environment provides the user with a biomechanics environment ^5^ (Akimov, 2020), with the goal being to control a human body to accomplish diverse locomotion tasks such as arm movements or different gait patterns.

Simulated closed-loop systems have, however, witnessed their biggest and maybe most popular breakthrough with the release of alphaGo (Silver et al., 2017), which beat the leading world champion in the game of Go. This was followed by alphaZero (Silver et al., 2018) and alphaStar (Vinyals et al., 2019) beating their respective leading world champion in chess, shogi, and most impressively Starcraft. The game of Go is known for its untraceable decision tree while Starcraft is a competitive online multi-player real-time strategy computer game, making the RL capabilities of these Deepmind engines truly impressive. What needs to be considered here, though, is that these engines were operating directly on machine-level code rather than through a layer of visual or motor abstraction, enabling them to operate far faster than biological real-time, without any added sensory-motor noise.

Similar approaches have been used to artificially master other games such as Mario, Quake III (Jaderberg et al., 2019), Dota 2 (OpenAI: et al., 2019), or a host of Atari games (Badia et al., 2020).

### 3.3. Simulators

Simulators play an important role in lowering the barriers to interaction with otherwise expensive or complicated hardware and can greatly aid the exploration and prototyping of new and novel neuromorphic hardware. Simulation can be applied directly to neuromorphic sensors and computing hardware, which can in turn be used to develop, test, and even characterise neuromorphic algorithms and approaches. Simulation also allows for the exploration of situations, scenarios, and environments that may be prohibitively difficult or pose technical challenges for real-world hardware.

Simulation techniques are already widely used in neuromorphic engineering. For example, simulation has been used to optimise existing event-based pixel designs (Remy, 2019) and to analyse and predict bottle-neck effects (Yang et al., 2017). Simulation can also allow for the rapid exploration of a vast number of potential scenarios, such as those found in real-world environments, and which would be impossible to physically test individually. Complex and hazardous scenarios are also expensive to emulate: for example, the pre-crash scenario used in designing of automotive vehicles can be tested with fake targets, but this restrains the evaluation to a single, very specific scenario, making the optimisation easy and leading to the issue of over-fitting (Segata and Cigno, 2019). Simulations enable us to explore a broader range of configurations in which there is direct access to the ground truth. This can also be used to augment and extend real-world datasets, such as for example Virtual KITTI (Gaidon et al., 2016) which extends the KITTI dataset (Geiger et al., 2013) to include simulated data for extreme driving conditions.

Simulators also enable the rapid exploration of the benefits offered by neuromorphic sensing when compared to conventional strategies, especially in cutting-edge challenges such as drone racing (Madaan et al., 2020) or pose estimation (Mueggler et al., 2017). Simulation further eliminates the need to calibrate several real sensors, which is itself a challenging and open question. Uncalibrated and uncharacterised sensors can add temporal and spatial errors through different acquisition speeds and unsynchronised clocks (Zhu et al., 2018).

Some simulators, such as Carla (Dosovitskiy et al., 2017), take advantage of highly sophisticated rendering engines developed and optimised for the gaming industry. These tools have been extended and adapted to emulate neuromorphic vision sensors and have been successfully used to simulate data for a number of challenging tasks. An early example of such an application was a simulated driving task in which the algorithm must control a robotic car and keep it on the road (Kaiser et al., 2017). As part of the Neurobotics project ^6^ it allows for the development of bio-inspired robots through simulations (Falotico et al., 2017). The project was built upon the Robotics Operation System(ROS) tool-chain (Quigley et al., 2009) and used a simulator known as Gazebo (Koenig and Howard, 2004), which emulates an event-driven pixel using rendered images discretised in time. This was followed by Event SIMulator (ESIM), which is perhaps the most widely used event-based vision simulator in the neuromorphic community (Mueggler et al., 2017; Rebecq et al., 2018). It provides a simulation of a more realistic pixel behaviour and implements a novel method to adapt the time resolution of the rendering as a function of the dynamics of the scene. It has been used to create annotated datasets (Rebecq et al., 2019), or to simulate novel pixel designs with multi-spectral sensitivities (Scheerlinck et al., 2019). More recently, we have developed an even more realistic event based vision sensor simulator (Joubert et al., 2021), which has been used to simulate characterising the materials on resident space objects with event based sensors (Jolley et al., 2021).

In the past, simulation models of event-based sensors have been used to extend computer vision open-loop datasets like classification (Gehrig et al., 2020) and as a means of converting conventional datasets to event-based ones (Gehrig et al., 2020). Whilst this approach has merits, it faces inherent limitations when applied for event-based vision systems as the high temporal resolution, a hallmark of event-based sensing, is artificially interpolated and subject to quantisation errors. The different sources of noise are also neglected, and this loss of information might be detrimental to building fully real-world applicable systems. Finally, some limitations remain as no simulator perfectly replicates the real world, and the quantity-quality trade-off of generated data, e.g., with respect to the level of detail in the simulation of the laws of physics, remains one of many unresolved limitations (Hu et al., 2021).

One of the most significant problems encountered with simulations relates to the often vast difference in difficulty between controlling a simulation and a physical system (Jakobi et al., 1995), with the main differences arising from the degree and nature of noise in the real-world system. We argue that this noise is not only inherent in neuromorphic systems, but perhaps even necessary to build functioning and robust algorithms and systems (Liang and Indiveri, 2019; Milde, 2019). The nature of noise in neuromorphic (and potentially biological systems) may be fundamentally different to how it is treated in conventional sensors and processing. Our efforts to mitigate this noise, either through post-processing or by designing systems that better approximate our idealised simulations, may have hindered our ability to deliver on the promises of neuromorphic algorithms and systems.

## 4. Novel Neuromorphic Closed-Loop Benchmarks

To close the gap between *perfect* simulations of the world and the *imperfect* reality we need to explore novel ways of building physically embedded closed-loop benchmarks and thus generate realistic training environments. This step towards closed-loop benchmarks will also spur and require the development of novel models of, and approaches to, machine and neuromorphic intelligence.

### 4.1. Looking Beyond Accuracy as a Single Benchmarking Metric

Accuracy is generally evaluated by calculating the difference between a desired high-level concept target (i.e., true object category) and the output of the model (i.e., the inferred object category). Accuracy alone does not encapsulate all performance metrics important in a real-world system. For example, closed-loop systems can have hard limitations placed on their response time, but the latency required to operate successfully is not captured by measures of performance accuracy. In order to address these restrictions we need to evaluate models beyond accuracy as a single benchmarking metric.

The majority of approaches to formulating an evaluation metric exclude training and execution time from the loss function and thus from the performance evaluation (Torralba and Efros, 2011). Similarly, power consumption, throughput and operations performed per unit time are not considered (Torralba and Efros, 2011). In addition there are other system evaluation metrics, such as racial or gender recognition biases or resiliency to adversarial attacks (Stock and Cisse, 2018).

Here, we propose to include these constraints implicitly in the benchmark's evaluation metric. Thus, the objective of a model competing on a physically embedded benchmark becomes to achieve the highest score with limited power consumption, unbiased data collection, limited throughput and a hard time constraint to react in biological or task-dependent real-time. This paradigm shift will spur the development of models which focus on closed-loop and predictive sensing and processing (Rao and Ballard, 1999; Moeys et al., 2016; Keller and Mrsic-Flogel, 2018), exploit spatio-temporal sparsity (Aimar et al., 2019) and are suited for novel real-time performing neuromorphic processing systems (Milde et al., 2017).

### 4.2. A Case Study for Event-Based Closed-Loop Benchmarks

Building on the needs and requirements identified for neuromorphic benchmarking systems, we have developed a set of required characteristics that are essential for creating benchmarking tasks that properly assess and quantify the performance of neuromorphic systems. These are:

**Evaluation Metrics:** The experimental setup should be capable of collecting critical information, such as power consumption and task performance, which is needed to evaluate the models.

**Closed-loop embodiment:** The benchmarking task should require at least one level of feedback. Therefore, the output, whether originating from early, intermediate, or late processing stages of the model should affect the input to the model, for example, by altering either perceptual parameters of the sensor, or the relative positioning of the agent and its sensors with respect to the environment.

**Complexity:** The environments should reflect the complexity that an agent can encounter in real-world scenarios and therefore include multiple possible states. The presence of noise, occlusions, and background clutter (or the equivalent noise and distractors in non-visual tasks) needs to be part of the environment if we desire to develop processing algorithms that are resilient to such effects. It is also important that the same environment be available for both the training and the testing environment.

**Accessibility and Latency:** The benchmarking task needs to be remotely accessible and have a clearly defined Application Programming Interface (API) to enable testing of different algorithms. The API should be capable of relaying and recording all the essential information from the experimental setup to the model and vice versa. The API needs to be open-source for transparency and needs to support different existing conventional and neuromorphic architectures. The API needs to operate at high-speed, with low latency, to allow algorithms to take full advantage of neuromorphic sensory-processing systems.

**Replicability:** The dynamics of the environment need to be able to be replicated. The experimental setup should be reliable enough to handle long trials and multiple runs with minimal deviation in performance. The setup has to sustain its behaviour over very long periods and produce reliable and repeatable results. As closed-loop benchmarks must evaluate applied algorithms in a consistent unbiased manner, they must, by necessity, exclude non-reproducible physical systems with non-ergodic behaviour.

The final point in this list implies that ideal closed-loop benchmarks cannot contain humans in the loop. However, a system which supports both, robot vs. robot and robot vs. human interaction, can be very interesting as the human opponent represents a source of *noise* which is informed but neither unbiased nor consistent. The introduction of a human opponent will also help in engaging non-experts in the discussion on the implications of research for the general public and will make it easier to convey the scientific efforts similarly to DeepMind's efforts with alphaGo.

**Figure 4:**
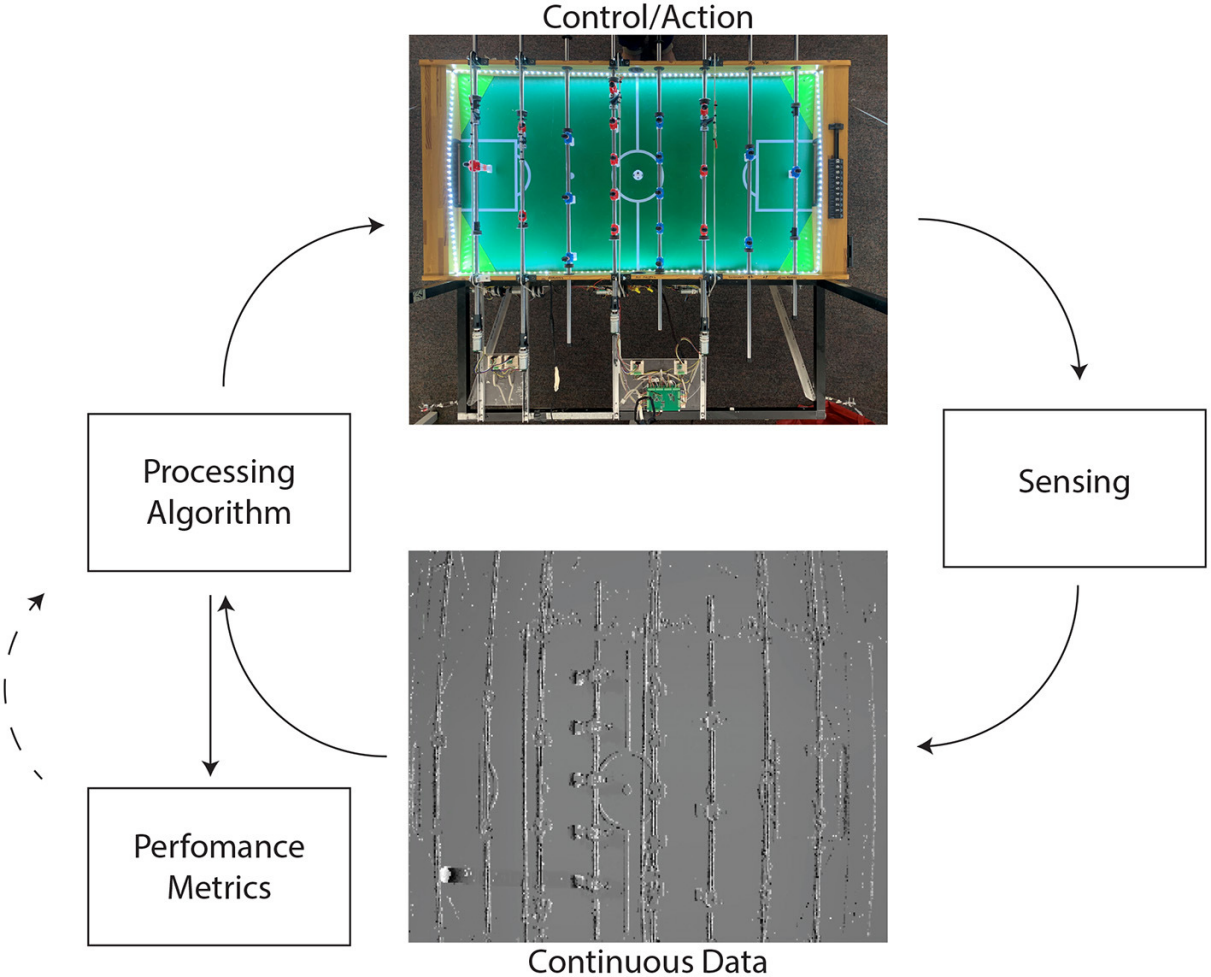
Schematic of the closed-loop robotic foosball setup.

To better define what such a novel, physically embedded closed-loop benchmark could look like, we will describe in the remainder of this article our efforts towards building a robotic foosball table. We started the design and development of the first iteration of a robotic foosball table for the 2019 Telluride Neuromorphic Engineering Workshop ^7^. The idea was simply that, if a human or algorithm can beat another human or artificial opponent, i.e., score more goals in a game, the winner is better at the task of table foosball, giving us a straightforward performance metric (see Figure 4).

The setup was a standard foosball table with one side controlled by the machine and the other side open for human play. A strip of non-flickering LEDs illuminated the surface of the table. The ball had no special markers on its surface, to help in differentiating it from other movements on the table. A neuromorphic event-based camera (Brandli et al., 2014) was mounted on top of the table looking directly down towards the table surface providing both regular sampled frames and asynchronously sampled events. Neuromorphic vision sensors are exquisite at picking up fast-moving objects against a stationary background, but the dynamic motion of the player rods by both contestants provides many distractions by obstructing the ball below them.

The machine had eight degrees of freedom to control the translational and rotational movements of the four rods on the machine side. The mechanics were developed to ensure fast movement of the players with low latency to match the speed of the ball. One way to interact with the environment was through direct access to the eight motors controlling the rods *via* a micro-controller, but a more abstract and simple level of control was provided by controlling the position of the players on the table.

The problem of building a table foosball controller can be approached in multiple ways; it can be treated as a compound task of tracking and decision making, or as an end-to-end reinforcement learning task. The fast and dynamic environment demands algorithms which are capable of real-time processing of the events from the (neuromorphic) vision sensor. Thus, the benchmark intrinsically requires real-time predictive inference for successful gameplay and greatly benefits from non-batched, online and continuous learning approaches. The reason for this is simple: if one wants to hit a ball it is of negligible importance where the ball has been in the past, it hardly matters where the ball is right now, but it truly matters where the ball is going to be when one wants to hit it.

In this system, the performance evaluation can mainly be the game score and power consumption. On the foosball system we propose, the power consumption is constraint by the hardware we make available, but the computational demands of different algorithms will still impact the effective power consumption of the system. Also, the robotic foosball table as a benchmark could be copied in different locations and could use different hardware with different power consumption limitations. Such game score driven evaluation has been sufficient for developing systems such as alphaGo (Silver et al., 2018), but for human designed algorithm development, additional feedback will be required. For this purpose, recordings of the system made with the neuromorphic event-based camera will be made available to the researcher.

The current prototype iteration of the robotic foosball table is not yet ideal as a benchmark for neuromorphic algorithms. In an ideal scenario, both sides should be controlled by an algorithm or network and the winner remains and can be contested by another algorithm or network. We are currently developing such a table, where both sides can be controlled by a robotic system as well as a software stack for allowing remote access of the benchmark through a web-based API. We expect this foosball setup will pose a good first benchmark for conventional and neuromorphic algorithms to test their capabilities in a closed-loop setting.

## 5. Concluding Remarks

In this article, we discussed the understandable reasons why the research community, whether neuromorphic or not, gravitates towards open-loop datasets to train and evaluate their artificially intelligent algorithms or networks. While models and hardware accelerators are being developed to ensure operational real-time performance during inference on such open-loop datasets, online training within a limited time and power budget is being neglected in these solutions. Alternatively, the closed-loop nature of Reinforcement Learning (RL) introduces a notion of online learning and decision making in models of machine intelligence. Conventional RL approaches introduce the requirement for operational real-time performance in inference, but not in training, nor do they address the issue of power consumption in their evaluation metrics. It appears that in most cases, the power consumption and real-time performance of both training and inference in models of machine intelligence are treated as afterthoughts, to be optimised afterwards using dedicated hardware accelerators or application-specific integrated circuit solutions.

The neuromorphic community has greatly benefited from the vast number of open-loop datasets and has often recreated and converted them for use in training neuromorphic algorithms and neural networks. However, the same is not true for closed-loop benchmarks, even though such benchmarks would play to the strengths of neuromorphic sensory-processing systems, i.e., low power consumption, high temporal resolution, distributed and local learning, robustness to noise, resilient processing due to parallel and redundant information processing pathways, and online unsupervised learning. The very essence of the event-based sensing and computing paradigm, that time represents itself, should enable neuromorphic algorithms and spiking neural networks to naturally implement feedback control loops in which time and its continuous representation can act as the unifying entity for perception, learning, and action. The neuromorphic community is, however, missing benchmark tasks that require recurrent and feedback heavy algorithms and networks. To enable testing this assumption, we described our efforts in building a closed-loop, physically embedded robotic foosball system to function as a benchmark. We expect that robotic foosball, or similar physically embedded closed-loop benchmarks, will be a crucial ingredient in advancing machine and neuromorphic intelligence to include the ability to perform time critical, informed decisions in noisy, ambiguous environments based on often partial information.

## Author Contributions

All authors listed have made a substantial, direct, and intellectual contribution to the work and approved it for publication.

## Funding

This project was funded by Western Sydney University's Strategic Research Initiative. Some of the authors were supported by AFOSR grant FA9550-18-1-0471.

## Conflict of Interest

The authors declare that the research was conducted in the absence of any commercial or financial relationships that could be construed as a potential conflict of interest.

## Publisher's Note

All claims expressed in this article are solely those of the authors and do not necessarily represent those of their affiliated organizations, or those of the publisher, the editors and the reviewers. Any product that may be evaluated in this article, or claim that may be made by its manufacturer, is not guaranteed or endorsed by the publisher.

## Footnotes

^1^ https://www.frontiersin.org/research-topics/3448/

^2^See https://www.frontiersin.org/research-topics/7663/active-sensing for example

^3^ https://gym.openai.com/envs/#atari

^4^ https://gym.openai.com/envs/#mujoco

^5^ http://osim-rl.stanford.edu/

^6^ https://neurorobotics.net

^7^ http://tellurideneuromorphic.org
